# Establishment of Recombinase-Aided Amplification Combined with Lateral Flow Dipstick for Rapid Detection of *Yersinia pestis* Based on the *pla* Gene

**DOI:** 10.3390/vetsci13101091

**Published:** 2026-10-14

**Authors:** Yunhan Zhou, Xue Yang, Ziling Ding, Qiuyu Fan, Xiaofang Yan, Haifeng Liu, Ziyao Zhou, Zhijun Zhong, Jialiang Xin, Guangneng Peng

**Affiliations:** 1 Key Laboratory of Animal Disease and Human Health of Sichuan, College of Veterinary Medicine, Sichuan Agricultural University, Chengdu 611130, China; yunhan_zhou@163.com (Y.Z.); yangx00112@163.com (X.Y.); 18228090550@163.com (Z.D.); fanqiuyuyuriki@163.com (Q.F.); 13550001726@163.com (X.Y.); 410140017@163.com (H.L.); zzhou@sicau.edu.cn (Z.Z.); zhongzhijun488@126.com (Z.Z.); 2 College of Veterinary Medicine, Northwest A&F University, Yangling 712100, China

**Keywords:** *Yersinia pestis*, recombinase-aided amplification (RAA), lateral flow dipstick (LFD), rapid detection, *pla* gene

## Abstract

This study establishes a rapid detection method for *Yersinia pestis* that combines recombinase-aided amplification with a lateral flow dipstick (RAA-LFD) and targets the *pla* gene. Based on the *pla* gene sequences of six representative strains from NCBI, three primer pairs (P-F1/R1, P-F2/R2, and P-F3/R3) and one RAA-nfo probe were designed. The RAA-LFD assay was constructed and evaluated for sensitivity, specificity, repeatability, and clinical applicability. Initial screening via polymerase chain reaction followed by agarose gel electrophoresis (PCR-AGE) showed P-F3/R3 produced a faint band, while P-F1/R1 and P-F2/R2 yielded bright, specific bands. RAA-LFD condition optimization demonstrated that: clear test lines (T lines) were observable at primer concentrations of 250–2000 nM, with the strongest signal at 2000 nM; the T line signal of P-F1/R1 remained stable at 35–41 °C, peaking slightly at 39 °C, whereas P-F2/R2 decreased markedly at 35 °C (*p* < 0.01); and the integrated optical density (IOD) of the T line increased with reaction time from 5 to 30 min, with a significant difference between 5 and 30 min (*p* < 0.001). The optimal reaction conditions were consequently determined as follows: primer pair P-F1/R1, a final primer concentration of 2000 nM, and incubation at 39 °C for 30 min. All five *Y. pestis* strains tested positive, while seven non-target strains and blank controls were negative, consistent with PCR results. Regarding sensitivity, the limit of detection (LOD) of RAA-LFD was 3.8 × 10^2^ copies/μL, approximately 10-fold lower than conventional PCR (3.8 × 10^3^ copies/μL). In the repeatability evaluation, intra-assay CVs were 1.79%, 0.59%, and 7.76%; inter-assay CVs were 1.75%, 2.35%, and 1.70%, all within 10%. Results for 130 clinical samples showed 100% agreement with PCR and qPCR. RAA-LFD established in this study provides a reliable, simple approach for on-site plague screening and primary laboratory diagnosis.

## Simple Summary

Plague is a life-threatening zoonotic disease caused by *Yersinia pestis*, which remains endemic in certain regions with limited laboratory infrastructure. In this study, we developed a rapid detection method combining recombinase-aided amplification with a lateral flow dipstick (RAA-LFD) targeting the *pla* gene of *Y. pestis*. The method operates under isothermal conditions and produces visible results on test strips without the need for sophisticated instrumentation. We evaluated its performance comprehensively and found that it exhibited excellent specificity, sensitivity, repeatability, and clinical applicability. This method improves the efficiency of plague diagnosis and provides a practical tool for on-site screening and primary care settings.

## 1. Introduction

*Yersinia*, a genus within the family Enterobacteriaceae, encompasses three species that pose significant threats to human health: *Yersinia pestis*, *Yersinia pseudotuberculosis*, and *Yersinia enterocolitica* [1]. As the type species of the genus, *Y. pestis* is a non-spore-forming, Gram-negative coccobacillus that lacks flagella, pili, and a capsule. It is transmitted primarily through bites from infected fleas and may also infect the host via respiratory droplets, direct contact, or other routes [1]. Plague, an acute infectious disease caused by *Y. pestis*, is designated by the World Health Organization (WHO) as one of the re-emerging infectious diseases under priority surveillance [2] and is characterized by rapid transmission, strong pathogenicity, and high mortality. According to the route of infection and clinical manifestations, plague is generally classified into three forms: bubonic, pneumonic, and septicemic plague, among which pneumonic plague is the most severe, with case fatality rates approaching 100% if not diagnosed and treated within 24 h of disease onset [1]. Historically, plague has caused three devastating pandemics, resulting in hundreds of millions of deaths [3]. To date, natural plague foci remain widely distributed across multiple countries and regions in Africa, Asia, South America, and North America, with the Democratic Republic of the Congo, Madagascar, and Peru reporting the highest numbers of cases [3,4]. During the annual epidemic season, typically from September to April of the following year, sporadic cases and local outbreaks continue to be reported, posing a sustained threat to public health security [5,6].

Early, rapid, and accurate etiological detection is critical to plague prevention and control, directly influencing patient prognosis and the timeliness of public health emergency responses. Currently, laboratory detection methods for *Y. pestis* primarily include bacterial isolation and culture, biochemical identification, immunological assays, and molecular biological techniques [7,8]. Bacterial isolation and culture, regarded as the reference standard for diagnosis, can provide viable bacterial information and support subsequent antimicrobial susceptibility testing. However, culture typically takes 2–5 days and imposes stringent requirements for sample transport (cold-chain preservation and prompt delivery) and laboratory biosafety level (BSL-3). In remote endemic foci, delayed sample delivery or improper preservation frequently results in an elevated false-negative rate [3]. Serological assays, such as F1 antigen-capture enzyme-linked immunosorbent assay (F1 antigen-capture ELISA) and anti-F1 antibody detection, are relatively simple to perform; however, the antibody window period, along with the possibility of false-positive results due to previous infection or vaccination, limits their utility in meeting the diagnostic demands of the acute phase [9,10].

The emergence of isothermal nucleic acid amplification technologies offers a promising alternative to address the aforementioned challenges. Among these, loop-mediated isothermal amplification (LAMP) has been successfully applied to detect *Y. pestis* by targeting the *caf1A* gene, with amplification completed within 30 min at a constant temperature of 65 °C and visual interpretation via color changes, demonstrating considerable potential for field application [11]. However, LAMP primer design requires recognition of eight distinct regions within the target sequence, imposing stringent constraints on target selection and sequence compatibility, thereby limiting its flexibility and general applicability. In contrast, recombinase-aided amplification (RAA) is an emerging technology that enables rapid amplification of nucleic acids under isothermal conditions at 37–42 °C [12]. The core components of the RAA reaction system include a recombinase, single-stranded DNA-binding protein (SSB), and a strand-displacing DNA polymerase. The recombinase forms nucleoprotein complexes with primers that actively search for and invade homologous sequences within the double-stranded DNA template, mediating strand unwinding; SSB stabilizes the displaced single-stranded regions and prevents primer dissociation; and the DNA polymerase extends the primers in a strand-displacement manner, achieving exponential amplification of the target sequence [12]. Detection of the amplified products can be accomplished by agarose gel electrophoresis, real-time monitoring with fluorescent probes, or visual interpretation using lateral flow dipsticks [13]. In recent years, RAA technology has been widely employed to detect various pathogens, including parasites, viruses, and bacteria [14,15,16]. Among detection formats, the RAA-LFD combined system, which integrates the high sensitivity of isothermal amplification with the naked-eye visualization advantage of lateral flow chromatography, has shown promise for rapid on-site pathogen screening [17].

**Figure 1:**
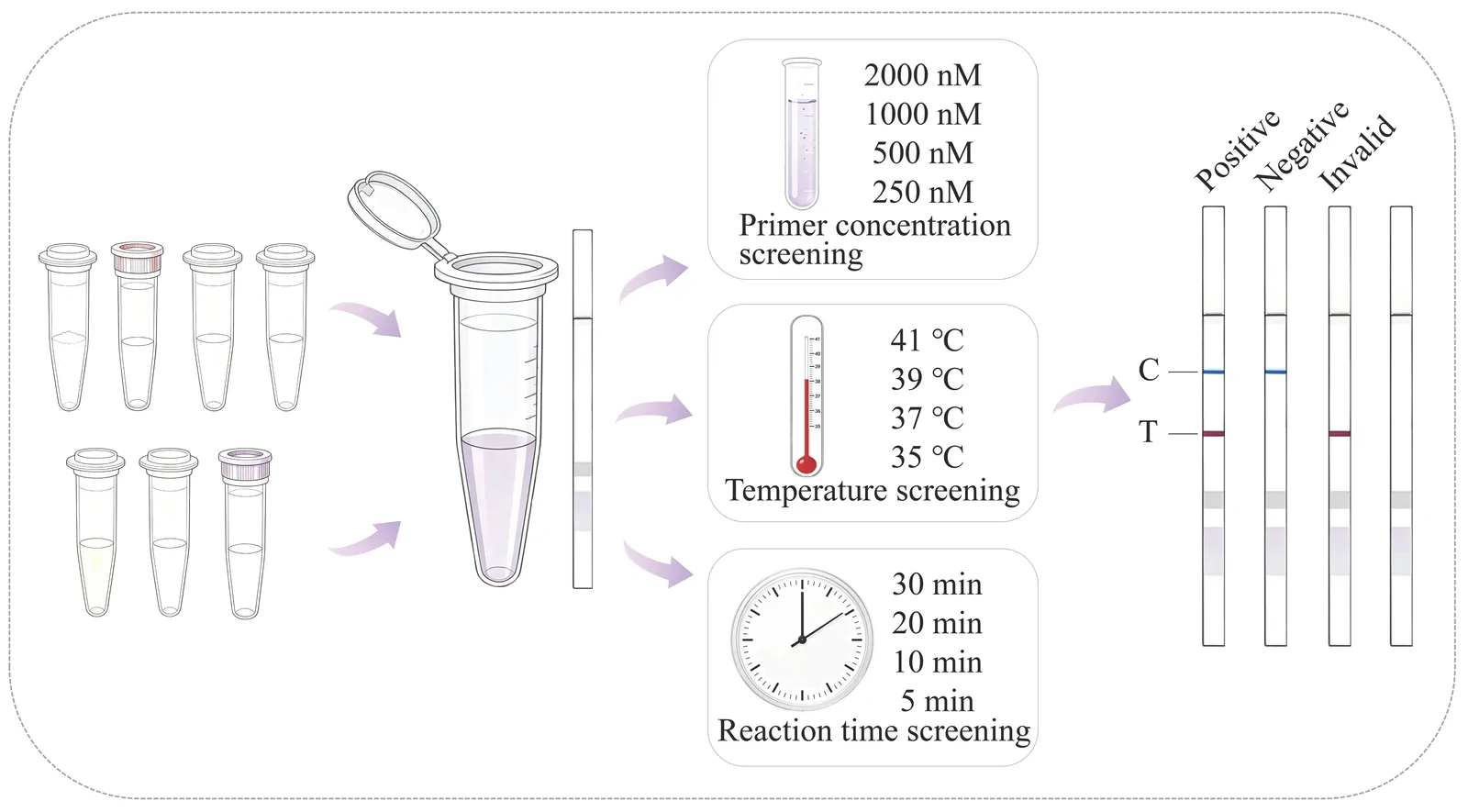
Flowchart of the technical workflow.

Regarding target gene selection, the plasminogen activator gene (*pla*) is located on the pPCP1 plasmid, which is unique to *Y. pestis*. This gene is present at a high copy number (150–200 copies per bacterium). It is abundantly expressed in bacterial cells, making it one of the most frequently used targets in PCR-based detection of *Y. pestis* [18]. In this study, we established an RAA-LFD rapid detection method targeting the *pla* gene of *Y. pestis*. After systematically optimizing reaction conditions, we comprehensively evaluated the method for analytical sensitivity, specificity, and repeatability and validated its practical detection performance on clinical samples, aiming to provide a technically simple, cost-effective, and reliable tool suitable for on-site rapid screening of plague and for diagnostic application in primary laboratories. The technical workflow is illustrated in Figure 1.

## 2. Materials and Methods

### 2.1. Bacterial Strains

In total, six bacterial strains were used in this study for methodological evaluation, including five *Y. pestis* strains (Otten), two other *Yersinia* species (*Y. pseudotuberculosis* CMCC53521 and *Y. enterocolitica* CNCC52203), and three non-*Yersinia* common pathogenic bacteria (*Escherichia coli* CMCC44102, *Klebsiella pneumoniae* CMCC46117, and *Salmonella Typhi* CMCC50071). All strains were obtained from the National Institute for Food and Drug Control (NIFDC), China.

### 2.2. Primer and Probe Design

Reference sequences of the *pla* gene were retrieved from the National Center for Biotechnology Information (NCBI) database, encompassing sequences from representative strains of diverse geographical origins, biovars, and phylogenetic lineages (accession numbers: GCF_000009065.1, KM880025.1, VEZU01000161.1, VWRY01000146.1, NZ_NYJS01000165.1, and NZ_JAKZDI010000113.1). We performed a multiple sequence alignment using SnapGene software 8.0 (Dotmatics, Boston, MA, USA) to assess nucleotide conservation and identify optimal target regions for primer and probe design. To verify the species specificity of the candidate regions, homology searches were conducted using the NCBI BLAST tool (https://blast.ncbi.nlm.nih.gov/Blast.cgi (accessed on 1 July 2026)) to systematically exclude sequences with high homology to non-target microorganisms, including closely related *Yersinia* species and other common enteric pathogens. Ultimately, we retained fragments exhibiting strong species specificity and high conservation for primer and probe design. Primers and probes were designed using Primer Express software 3.0.1 (Thermo Fisher Scientific, Waltham, MA, USA) and synthesized by Zhongce Biotech Co., Ltd. (Hangzhou, China). This study designed three pairs of candidate primers and one RAA-nfo probe, with their sequences listed in Table 1.

The labeling strategy for the primers and probe was as follows: the downstream primer was labeled with biotin at the 5′ end to enable specific capture on the test line (T line) of the lateral flow dipstick through streptavidin–biotin interaction; the probe was labeled with 6-carboxyfluorescein (6-FAM) at the 5′ end to serve as the recognition site for colloidal gold-conjugated anti-FAM antibodies; a tetrahydrofuran residue (THF, i.e., dSpacer) was introduced internally as the cleavage site for endonuclease IV (nfo); and the 3′ end of the probe was blocked with a C3 spacer to prevent extension by DNA polymerase, thereby ensuring efficient nfo cleavage during amplification.

To select the primer pair with optimal amplification performance, we preliminarily validated the three candidate primer pairs by polymerase chain reaction followed by agarose gel electrophoresis (PCR-AGE). The PCR amplification protocol was performed according to the method described by Zhao et al. [19], with the following conditions: pre-denaturation at 95 °C for 5 min; 30 cycles of denaturation at 95 °C for 1 min, annealing at 62 °C for 30 s, and extension at 72 °C for 1 min; and a final extension at 72 °C for 10 min. The primer pair that exhibited strong specificity, bright bands, and satisfactory amplification efficiency was selected for subsequent construction and optimization of the RAA-LFD detection system.

### 2.3. RAA-LFD Assay

Recombinase-aided amplification (RAA) was performed using an RAA-nfo kit (dipstick type; Zhongce Genetic Biotechnology, Hangzhou, China). Each 50 μL reaction contained 25 μL Buffer A, 12.9 μL nuclease-free water, 2 μL each of upstream and downstream primers (final concentration 80 nM each), 0.6 μL probe (final concentration 24 nM), 5 μL DNA template, and 2.5 μL Buffer B. A master mix of nuclease-free water, Buffer A, primers, and probe was prepared for all samples, mixed thoroughly, and aliquoted into reaction tubes containing the lyophilized reagent. A DNA template was added to each tube, and Buffer B was pipetted onto the inner wall of the cap. After gentle mixing and brief centrifugation (2000× *g*, 10 s), reactions were incubated at 39 °C for 30 min in a water bath. For lateral flow detection, 50 μL of amplified product was diluted in 300 μL PBS (pH 7.4), and the sample pad of a universal nucleic acid dipstick was immersed in the solution and allowed to develop at room temperature for 10 min. A result was considered positive when both the test (T) and control (C) lines appeared, negative when only the C line appeared, and invalid when the C line was absent. Each run included a positive control (*Y. pestis* standard DNA) and a negative control (nuclease-free water).

### 2.4. Optimization of the RAA-LFD Reaction System

To achieve optimal reaction efficiency of the RAA-LFD assay, the primer concentration, reaction temperature, and reaction time were systematically optimized in this study. For primer concentration optimization, with a fixed probe concentration (2 μM), the final primer concentrations were set at 250 nM, 500 nM, 1000 nM, and 2000 nM. The color intensity and specificity of the LFD test bands were used as evaluation criteria to determine the optimal primer concentration. For reaction temperature optimization, under the optimal primer concentration, the reaction mixtures were incubated in constant-temperature water baths at 35 °C, 37 °C, 39 °C, and 41 °C, and the amplification efficiency at each temperature was compared to identify the optimal reaction temperature. For reaction time optimization, at the optimal primer concentration and reaction temperature, incubation times of 5 min, 10 min, 20 min, and 30 min were tested, and the optimal reaction time was defined as the shortest time required to obtain clear, stable positive bands. In each optimization experiment, positive and negative templates were tested in parallel, with three replicates per group.

### 2.5. Specificity Evaluation

Genomic DNA of the six bacterial strains, described in Section 2.1, was used as templates to evaluate the specificity of the RAA-LFD assay for *Y. pestis* detection. The tested strains included *Y. pestis* strains (Otten), two other *Yersinia* species (*Y. pseudotuberculosis* and *Y. enterocolitica*), and three non-*Yersinia* enteric pathogens (*E. coli*, *K. pneumoniae*, and *S. Typhi*). DNA extraction was carried out completely in accordance with the instructions provided by the reagent manufacturer (DP304, Tiangen, Beijing, China). Nuclease-free water was used as the blank negative control. All samples were tested using the RAA-LFD assay established in this study, with conventional PCR (primers and reaction conditions as described in Section 2.2) performed in parallel to compare the consistency of results between the two methods.

### 2.6. Sensitivity Evaluation

To evaluate the analytical sensitivity of the RAA-LFD assay, a recombinant plasmid carrying the target sequence of the *Y. pestis pla* gene (or genomic DNA of *Y. pestis*, as appropriate) was used as the template. The mass concentration of the template was converted to copy number concentration using the following formula: copies/μL = [DNA concentration (ng/μL) × 6.022 × 10^23^]/[genome size (bp) × 650 × 10^9^], where 650 represents the average molecular weight of one base pair of double-stranded DNA and the genome size of *Y. pestis* is approximately 4.6 × 10^6^ bp (4.6 Mb). Following quantification with a NanoDrop spectrophotometer (Thermo Fisher Scientific, Waltham, MA, USA), the template was serially diluted tenfold in nuclease-free water to generate standard dilutions ranging from 3.8 × 10^5^ to 3.8 × 10^1^ copies/μL. Nuclease-free water was used as the negative control. RAA-LFD detection was performed under the optimal conditions determined in Section 2.4, with conventional PCR (using the same primer pair without the probe) conducted in parallel to compare the limits of detection (LODs) between the two methods. The LOD was defined as the lowest concentration at which positive results were obtained in at least two out of three independent replicates.

### 2.7. Repeatability and Reproducibility Evaluation

Precision was evaluated under optimized RAA-LFD reaction conditions using *Y. pestis* DNA templates at three concentrations: (1 × 10^5^, 1 × 10^4^, and 1 × 10^3^ copies/μL). Intra-assay repeatability was determined by testing three replicates per concentration in a single run, with the positive detection rate and coefficient of variation (CV) of T line color intensity recorded. Inter-assay reproducibility was assessed by repeating the experiments on three separate days with different operators to examine batch-to-batch stability. Nuclease-free water was included as the negative control. T line color intensity was semi-quantitatively measured using ImageJ 1.8.0 software (National Institutes of Health, Bethesda, MD, USA) based on integrated optical density (IOD). CV was calculated using the following formula: CV (%) = (standard deviation/mean) × 100%.

### 2.8. Clinical Sample Collection and Detection

A total of 130 wild rodent spleen samples were collected from natural plague foci across five provincial-level administrative regions in China, comprising 27 from Heilongjiang Province, 15 from Jilin Province, 12 from Liaoning Province, 53 from Sichuan Province, and 23 from the Guangxi Zhuang Autonomous Region. Sample collection was approved by the Ethics Committee of the affiliated institution and complied with relevant national regulations on wildlife sampling. Genomic DNA was extracted from each sample using a commercial bacterial DNA extraction kit (DP304; Tiangen, Beijing, China) according to the manufacturer’s instructions. All extracted DNA samples were tested in parallel by the RAA-LFD assay developed in this study, conventional PCR (primer pair P-F1/R1), and a previously described real-time quantitative PCR (qPCR) assay [20]. Each PCR was performed in a 25 μL reaction mixture prepared according to the manufacturer’s instructions (P222-01; Vazyme Biotech, Nanjing, China). Conventional PCR was performed as follows: initial denaturation at 95 °C for 5 min; 30 cycles of denaturation at 95 °C for 1 min, annealing at 62 °C for 30 s, and extension at 72 °C for 1 min; followed by a final extension at 72 °C for 10 min.

### 2.9. Statistical Analysis

All experiments were independently repeated three times. The T line color intensity of the LFD strips was analyzed semi-quantitatively using ImageJ, and IOD values were measured and recorded. Statistical analyses were performed using GraphPad Prism 10.1.2 software (GraphPad Software, Boston, MA, USA). The effects of different primer concentrations, reaction temperatures, and reaction times on amplification efficiency were compared using one-way analysis of variance (ANOVA). Intra-assay and inter-assay repeatability were assessed by calculating CV values. *p* < 0.05 was considered statistically significant.

## 3. Results

### 3.1. Primer Validation and Optimization of the RAA-LFD Reaction System

**Figure 2:**
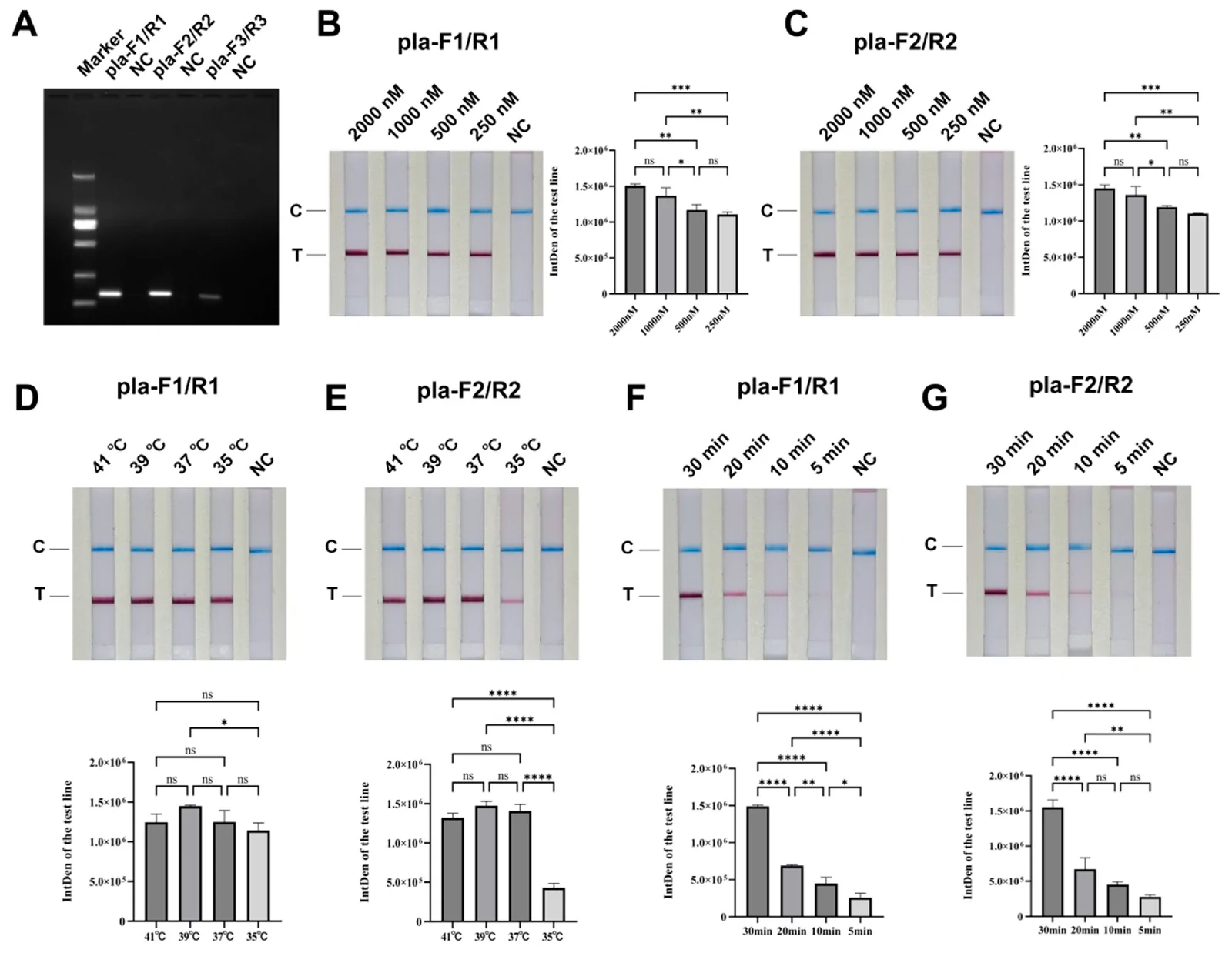
Optimization of reaction conditions for the RAA-LFD assay for *Y. pestis* detection. Data are presented as mean ± SD. * *p* < 0.05, ** *p* < 0.01, *** *p* < 0.001, **** *p* < 0.0001 (one-way ANOVA), ns = not significant. (**A**) PCR-AGE validation of three pairs of candidate primers. (**B**) Optimization of primer concentration for P-F1/R1 (250 nM, 500 nM, 1000 nM, and 2000 nM). (**C**) Optimization of primer concentration for P-F2/R2. (**D**) Optimization of reaction temperature for P-F1/R1 (35 °C, 37 °C, 39 °C, and 41 °C). (**E**) Optimization of reaction temperature for P-F2/R2. (**F**) Optimization of reaction time for P-F1/R1 (5 min, 10 min, 20 min, and 30 min). (**G**) Optimization of reaction time for P-F2/R2. Marker: DNA molecular weight marker DL 2000; NC: negative control.

In this study, three pairs of candidate primers (P-F1/R1, P-F2/R2, and P-F3/R3) and one RAA-nfo probe were designed targeting the *pla* gene of *Y. pestis*. The primer amplification performance was preliminarily validated by PCR-AGE. As shown in Figure 2A, all three primer pairs yielded specific bands of the expected sizes without nonspecific amplification; among them, P-F1/R1 and P-F2/R2 produced relatively bright and distinct bands, whereas P-F3/R3 exhibited a notably weaker band intensity. Accordingly, P-F1/R1 and P-F2/R2 were selected for further optimization. With the probe concentration and other reaction conditions held constant, the final primer concentrations for RAA-LFD detection were set to 250 nM, 500 nM, 1000 nM, and 2000 nM. As shown in Figure 2B,C, both primer pairs yielded clear T line signals across the concentration range of 250–2000 nM, and the IOD values of the T lines were positively correlated with primer concentration, with signal intensity gradually diminishing as concentration decreased. Taking both detection signal intensity and reagent cost into consideration, 2000 nM was ultimately selected as the optimal primer concentration.

At the optimal primer concentration, the RAA-LFD assay was assessed across four temperature gradients (35 °C, 37 °C, 39 °C, and 41 °C). As depicted in Figure 2D,E, the P-F1/R1 primer pair generated stable T line signals throughout the 35–41 °C range, accompanied by minimal IOD variation. Although the signal peaked marginally at 39 °C, the differences among the temperature groups were not statistically significant (*p* > 0.05). The overall trend for the P-F2/R2 primer pair was similar to that of the P-F1/R1 primer pair; however, a marked reduction in T line signal intensity was observed at 35 °C (*p* < 0.01), indicating poorer tolerance to low temperatures. Given that P-F1/R1 demonstrated superior temperature adaptability, particularly in maintaining stable amplification efficiency at lower temperatures, whereas P-F2/R2 showed a significant performance decline at 35 °C, P-F1/R1 was selected as the primer pair for all subsequent experiments, and 39 °C was determined as the optimal reaction temperature.

Using the P-F1/R1 primer pair at 2000 nM and a reaction temperature of 39 °C, the effect of reaction time on the detection signal was further investigated. Four reaction durations were tested: 5, 10, 20, and 30 min. As illustrated in Figure 2F,G, the IOD values of the T line increased markedly with prolonged reaction time, with the strongest signal observed at 30 min, followed by 20 min; the signal at 10 min was considerably weaker, and the signal at 5 min was the faintest, demonstrating a highly significant difference compared with the 30 min group (*p* < 0.001). To ensure both detection sensitivity and stability, 30 min was ultimately adopted as the optimal reaction time.

### 3.2. Specificity Analysis

**Figure 3:**
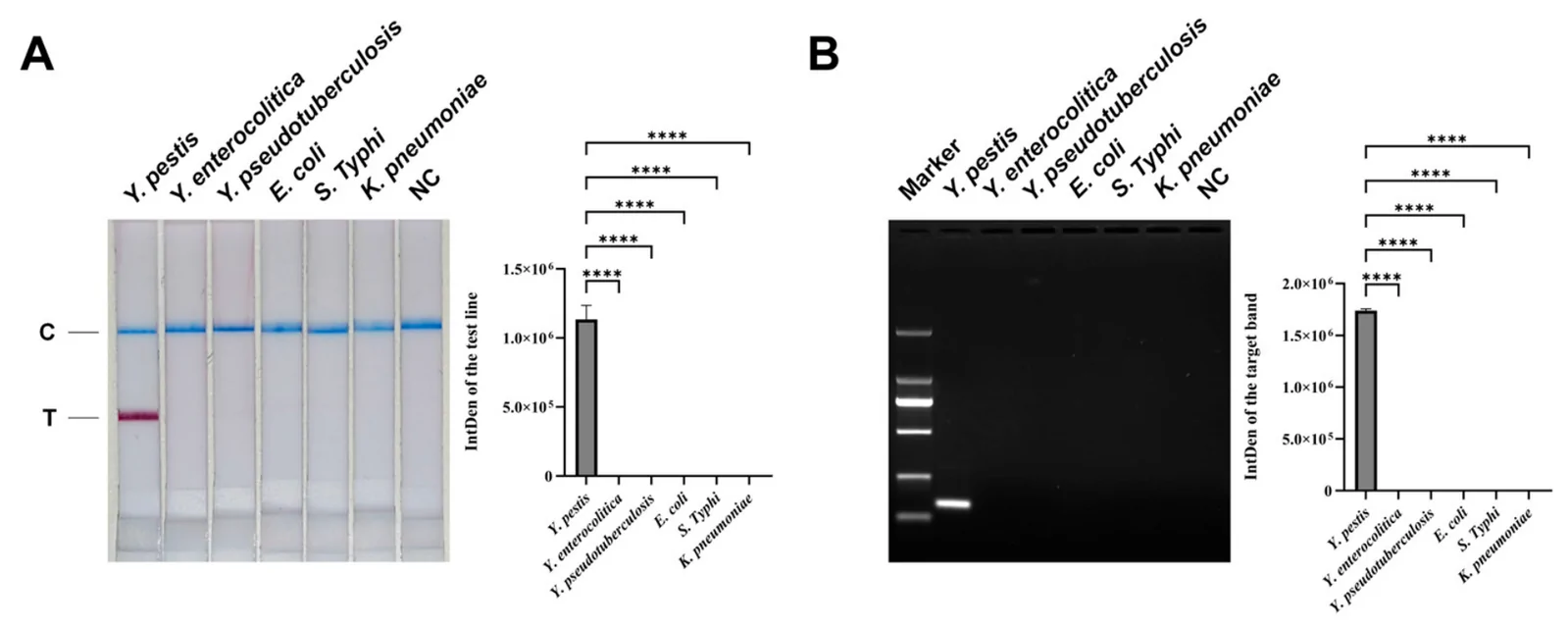
Specificity of the RAA-LFD assay for *Y. pestis* detection. Data are presented as mean ± SD. **** *p* < 0.0001 (one-way ANOVA). (**A**) RAA-LFD strip test results and corresponding T line IOD values using genomic DNA templates from *Y. pestis*, *Y. enterocolitica*, *Y. pseudotuberculosis*, *E. coli*, *S. Typhi*, and *K. pneumoniae*. (**B**) PCR-AGE results and corresponding band IOD (selected area × average gray value) for the same templates. Marker: DNA molecular weight marker DL 2000; NC: negative control.

Under the optimized reaction conditions (primers P-F1/R1 at 2000 nM, 39 °C, 30 min), the specificity of the RAA-LFD assay was evaluated using genomic DNA from five *Y. pestis* strains, two other *Yersinia* species (*Y. pseudotuberculosis* and *Y. enterocolitica*), three non-*Yersinia* enteric pathogens (*E. coli*, *K. pneumoniae*, and *S. Typhi*), and nuclease-free water as the blank negative control. As shown in Figure 3A, all five *Y. pestis* strains yielded clearly visible both T lines and C lines (positive results). In contrast, the other five non-target strains and the negative control exhibited only a single C line (negative results) and showed no cross-reactivity. Conventional PCR was performed in parallel for comparison, and the results were in complete agreement with those of the RAA-LFD assay: specific amplification bands were observed exclusively for *Y. pestis*, with no amplification detected for any of the other strains or the negative control (Figure 3B).

### 3.3. Sensitivity Analysis

**Figure 4:**
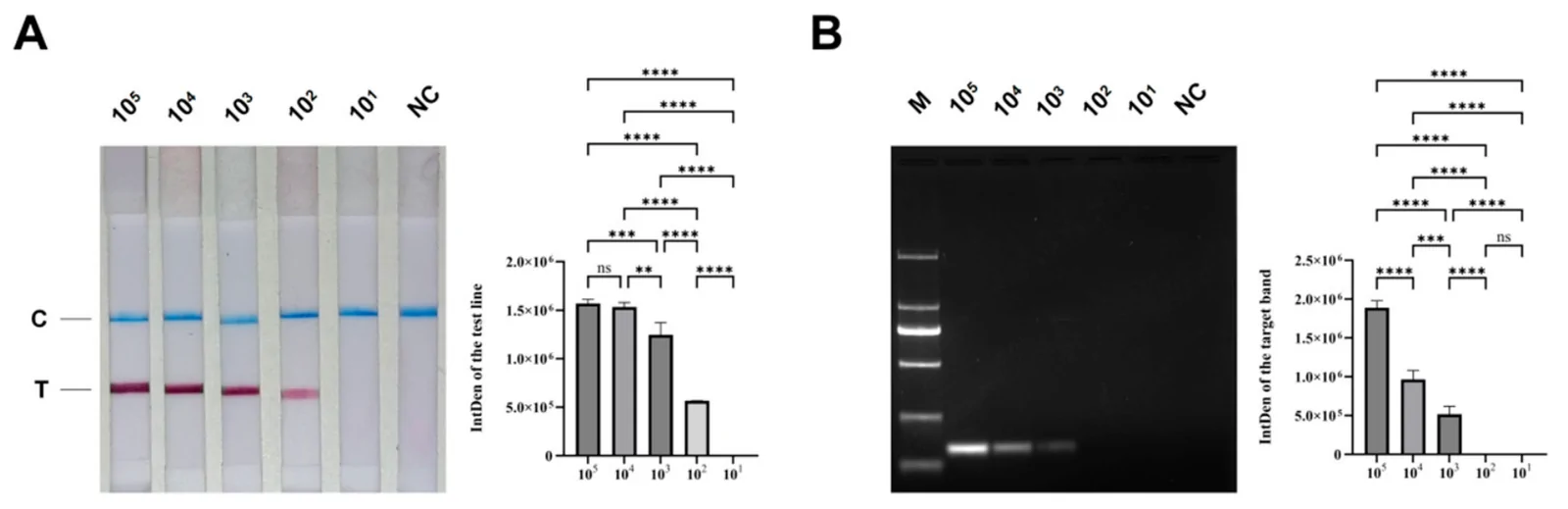
Sensitivity of the RAA-LFD assay for *Y. pestis* detection. Data are presented as mean ± SD. ** *p* < 0.01, *** *p* < 0.001, **** *p* < 0.0001 (one-way ANOVA), ns = not significant. (**A**) RAA-LFD detection results and corresponding T line IOD values for serial concentrations of *Y. pestis* DNA (3.8 × 10^5^–3.8 × 10^1^ copies/μL). (**B**) PCR-AGE detection results and corresponding band IOD values for the same concentration gradients. Marker: DNA molecular weight marker DL 2000; NC: negative control.

Genomic DNA of *Y. pestis* was serially diluted tenfold to prepare standard dilutions ranging from 3.8 × 10^5^ to 3.8 × 10^1^ copies/μL, with nuclease-free water used as the negative control. RAA-LFD detection was performed under the optimized reaction conditions, and the results were compared in parallel with those of conventional PCR. The T line color intensity of the RAA-LFD strips and the brightness of the PCR amplification bands gradually diminished as template copy numbers decreased. The RAA-LFD assay yielded a clearly detectable positive band at 3.8 × 10^2^ copies/μL, whereas no T line signal was observed at 3.8 × 10^1^ copies/μL (Figure 4A). PCR-AGE detected the target band at 3.8 × 10^3^ copies/μL but failed to produce a visible band at 3.8 × 10^2^ copies/μL (Figure 4B).

### 3.4. Repeatability Analysis

**Figure 5:**
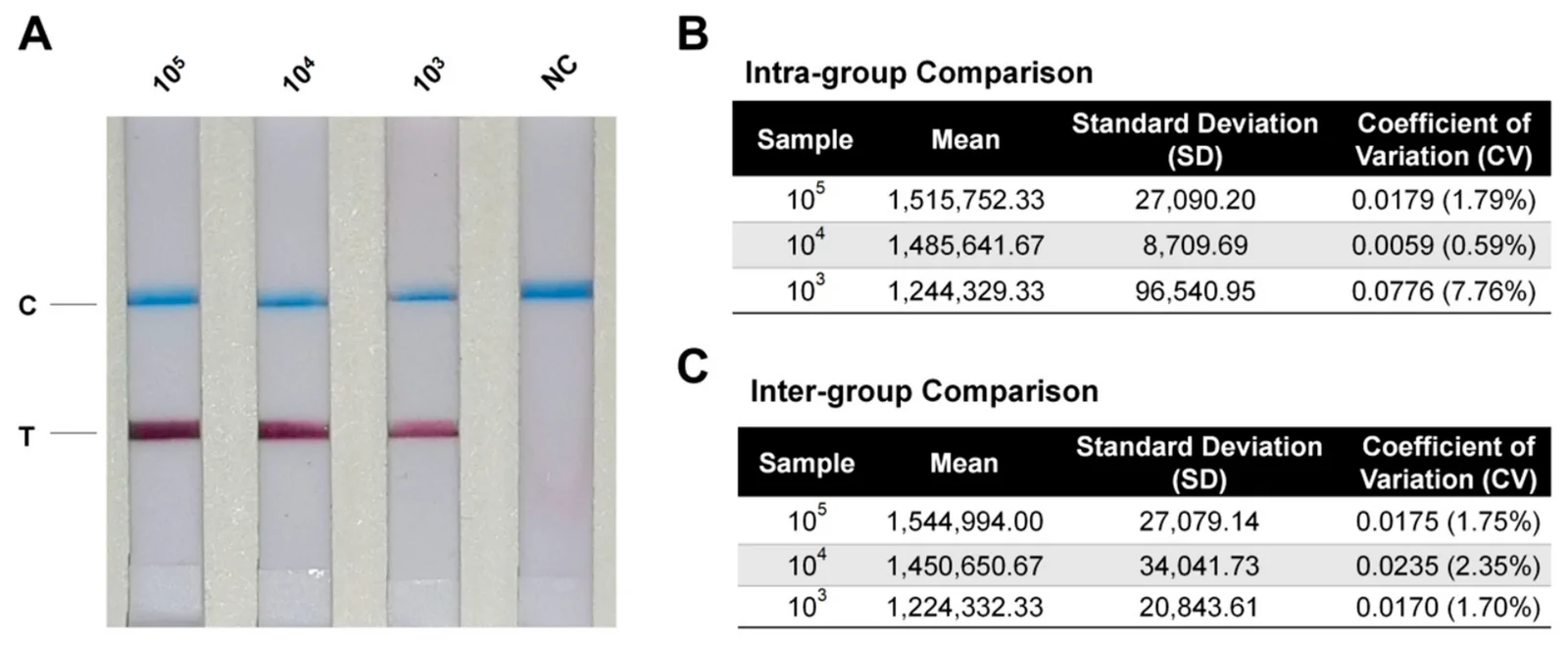
Repeatability of the RAA-LFD assay for *Y. pestis* detection. (**A**) RAA-LFD strip test results for different concentrations of *Y. pestis* DNA templates (3.8 × 10^5^, 3.8 × 10^4^, and 3.8 × 10^3^ copies/μL). (**B**) Standard deviation and coefficient of variation in T line IOD values for intra-assay repeatability. (**C**) Standard deviation and coefficient of variation in T line IOD values for inter-assay reproducibility.

Under the optimized RAA-LFD reaction conditions, three concentrations of *Y. pestis* DNA template—high, medium, and low (3.8 × 10^5^, 3.8 × 10^4^, and 3.8 × 10^3^ copies/μL)—were selected for precision evaluation. For intra-assay repeatability, the coefficients of variation (CVs) of the T line IOD values were 1.79%, 0.59%, and 7.76%, respectively (Figure 5A,B). For inter-assay reproducibility, the CVs of the T line IOD values for each concentration were 1.75%, 2.35%, and 1.70%, respectively (Figure 5C).

### 3.5. Clinical Sample Detection

**Figure 6:**
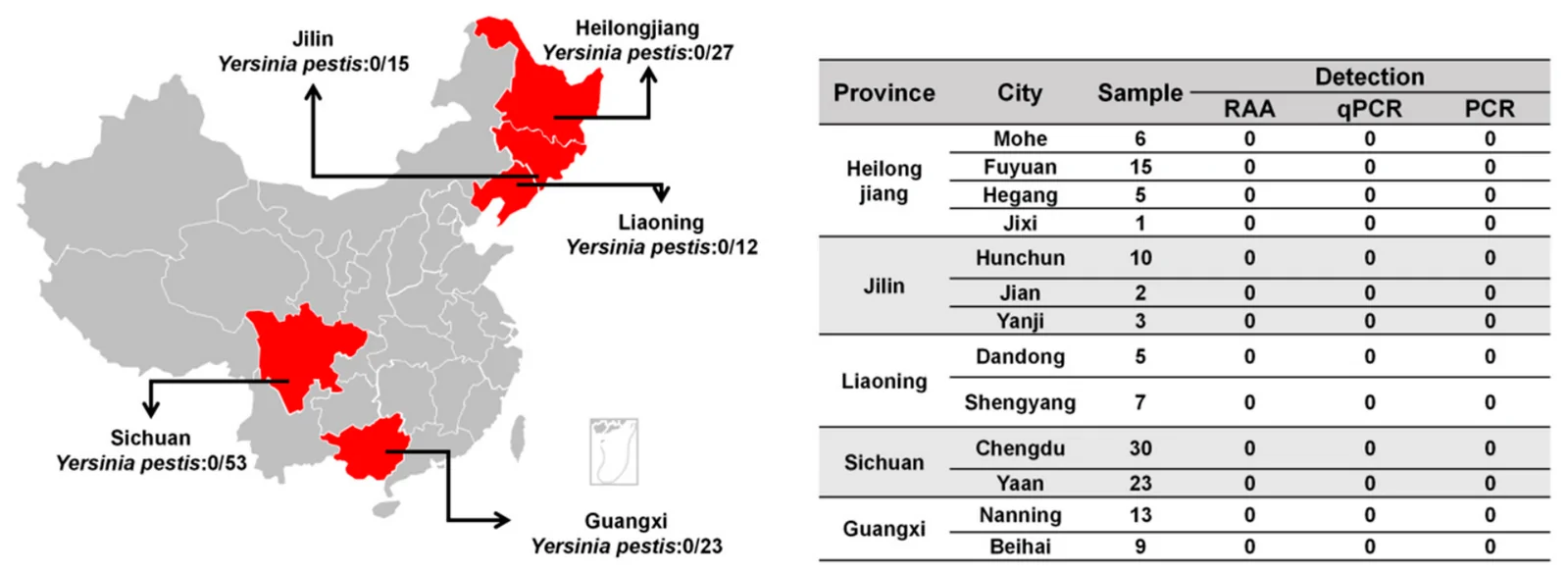
Clinical sample evaluation of the RAA-LFD assay for *Y. pestis* detection.

To validate the practical application value of the RAA-LFD assay, a total of 130 wild rodent spleen samples collected from five provincial-level administrative regions in China (Heilongjiang, Jilin, Liaoning, Sichuan, and Guangxi Zhuang Autonomous Region) were tested in parallel using the RAA-LFD method, conventional PCR, and qPCR. The results demonstrated complete concordance among the three detection methods, with no positive signals for *Y. pestis* detected in any of the 130 clinical samples (Figure 6). The overall concordance rate among the three methods was 100%.

## 4. Discussion

*Y*. *pestis*, the causative agent of plague, is classified by the World Health Organization (WHO) as an internationally quarantinable pathogen, and its prevention and control have long been prioritized within global infectious disease surveillance systems [21]. Plague is a typical zoonotic disease, with rodents serving as the primary reservoir hosts and fleas acting as vectors for enzootic transmission; humans become infected through contact with infected animals or through the bites of infective fleas [22]. Given its high pathogenicity, rapid transmission potential, and the historical lessons of devastating pandemics, the development of rapid detection systems applicable to diverse scenarios has remained a core requirement in the field of plague prevention and control.

Currently, laboratory diagnosis of plague relies primarily on three major categories of methods: etiological culture, serological assays, and molecular biological techniques. Bacterial isolation and culture, in conjunction with microscopy, remain the “gold standard” for definitive diagnosis [23]. However, this approach requires a prolonged culture period, strict BSL-3 laboratory conditions, and well-trained personnel. Moreover, in cases of delayed sample transport or improper preservation, the detection rate of viable bacteria decreases considerably, rendering this method unsuitable for on-site rapid screening and early warning. Serological assays, such as F1 antigen-capture ELISA, are relatively simple to perform; however, their performance is susceptible to fluctuations in antigen expression levels, host immune status, and cross-reactions among species, leading to risks of false-negative results during the window period and false-positive outcomes [24]. Among molecular biological methods, PCR and qPCR have become important tools for laboratory diagnosis of plague owing to their high sensitivity and specificity. Nevertheless, their dependence on precise thermal cyclers, stable power supplies, and trained operators limits their applicability in primary laboratories, field settings, and resource-limited regions.

In this study, we established a rapid RAA-LFD assay targeting the *pla* gene of *Y. pestis* and systematically optimized the key reaction parameters, including primer concentration, reaction temperature, and reaction time. The *pla* gene is carried on the multicopy pPCP1 plasmid (approximately 6–18 copies per cell), a considerably higher copy number than that of *caf1* on the low-copy pMT1 plasmid (1–2 copies per cell) or that of single-copy chromosomal markers (e.g., 3a, IS1541, and gyrB). For rapid on-site screening, where analytical sensitivity is the foremost priority, this multicopy target confers a clear advantage; moreover, *pla* is a recognized presumptive diagnostic target in the CDC plague case definition, further supporting its suitability for this application.

Regarding specificity, genomic DNA from two *Yersinia* species (*Y. pseudotuberculosis* and *Y. enterocolitica*) and three non-*Yersinia* enteric pathogens (*E. coli*, *K. pneumoniae*, and *S. Typhi*) were used as templates for control. The selection of these strains was based on the following considerations. First, *Y. pseudotuberculosis* and *Y. enterocolitica* belong to the same genus as *Y. pestis*, with *Y. pseudotuberculosis* being the most closely related species phylogenetically [25]; moreover, *Y. enterocolitica* infection has been shown to induce cross-protective immunity against *Y. pestis* [26]. Both species therefore serve as essential near-neighbor interference controls for specificity validation. Second, proteomic microarray analysis by Keasey et al. [27] demonstrated extensive antibody cross-reactivity between *Y. pestis* and various Gram-negative bacteria, including *E. coli* and *S. Typhi*; the inclusion of these species as controls confirmed that the present method effectively avoided such immunological cross-interference. Third, pneumonia caused by *K. pneumoniae* presents with clinical manifestations highly similar to those of pneumonic plague [28], posing a risk of misdiagnosis in clinical practice; the inclusion of this species in the specificity evaluation further confirmed the method’s ability to differentiate these pathogens at the etiological level accurately. Both RAA-LFD and PCR assays were performed for *Y. pestis* detection, and the results consistently demonstrated that only the *Y. pestis* DNA templates yielded positive amplification, indicating that the RAA-LFD assay exhibits excellent specificity for *Y. pestis*, with no cross-reaction or false-positive amplification.

Regarding analytical sensitivity, the LOD of the RAA-LFD assay was 3.8 × 10^2^ copies/μL, approximately 10-fold lower than that of conventional PCR (3.8 × 10^3^ copies/μL). Compared with recently reported isothermal amplification assays for *Y. pestis*, Feng et al. [29] developed a triplex-RPA system with a sensitivity of 1 copy/μL; Euler et al. [30] reported an RPA assay with an LOD of approximately 5–16 genome copies; and You et al. [31] established an RPA–Cas12a–UPTLFA platform targeting the *pla* gene with an LOD of 3 aM (approximately 1.8 copies/μL), albeit with a relatively complex workflow requiring approximately 1.3 h. Although the analytical sensitivity of our assay is lower than that of these methods, it offers a simpler workflow at substantially lower cost and—more importantly—remains sufficient for clinical screening, as supported by the following considerations. Blood cultures from septicemic plague patients can yield colony counts of up to 4 × 10^7^ CFU/mL [32], and the threshold for detectable bacteremia in pneumonic plague models reaches 6.4 × 10^4^–1.52 × 10^6^ CFU/g of blood (approximately equivalent to CFU/mL) [33]. Likewise, in a clinical evaluation of 149 patients with suspected bubonic plague in Madagascar, *pla* real-time PCR was positive in 120 lymph-node aspirates [34], and detection of *Y. pestis*-specific DNA by PCR is itself accepted as presumptive laboratory evidence in the CDC case definition [35]. Given that each bacterial cell harbors approximately 6–18 copies of the *pla* gene, the LOD of the present assay corresponds to roughly 2–6 × 10^4^ CFU/mL—below the bacteremic thresholds of acute pneumonic plague and well below the pathogen loads observed in acute infections. Furthermore, the entire assay is completed within approximately 40 min (30 min of amplification and 10 min of lateral flow development), and results can be interpreted visually without instrumentation. We therefore believe that the RAA-LFD platform adequately meets the requirements for rapid presumptive screening of plague at the point of care and in the field.

To evaluate repeatability and reproducibility, precision was assessed using three concentration gradients of *Y. pestis* DNA—high, medium, and low (3.8 × 10^5^, 3.8 × 10^4^, and 3.8 × 10^3^ copies/μL). The intra-assay CVs ranged from 0.59% to 7.76%, and the inter-assay CVs ranged from 1.70% to 2.35%, with all overall CVs falling within 10%. Cheng et al. [36] established a duplex RAA-LFD assay for detecting *Mycobacterium tuberculosis* and *Mycobacterium avium* complex, reporting inter-assay CVs of 2.16% and 2.05% for M. tuberculosis and 3.10% and 2.20% for *M. avium* complex at medium and low template concentrations, respectively, with overall CVs below 5%. Zhang et al. [37] performed independent RAA-LFA experiments for African swine fever virus at different template concentrations, yielding CVs of 2.73% for strong positive (10^5^ copies/μL), 4.01% for moderate positive (10^3^ copies/μL), 10.45% for weak positive (10^1^ copies/μL), and 8.76% for the negative control, indicating good repeatability except for weak positive samples. In comparison, the inter-assay CVs obtained in the present study (1.70–2.35%) are relatively low, suggesting favorable control of systematic error across different experimental batches and reliable operational reproducibility. The intra-assay CVs (0.59–7.76%) are also generally low. The upper limit of the CV range (approximately 8%) is attributable to the inclusion of three concentration gradients spanning a broad dynamic range; the weak colorimetric signal at the low concentration (3.8 × 10^3^ copies/μL) is inherently more susceptible to fluctuations in amplification efficiency, which reasonably accounts for the higher CV at this level. This observation is consistent with the findings of Zhang et al. [37], in which the weak-positive samples in their RAA-LFA study exhibited a higher CV (10.45%) than the strong- and moderate-positive groups. Notably, an overall CV of less than 10% is considered acceptable in molecular diagnostics and does not compromise the method’s clinical screening utility. Overall, the proposed method is well-suited as a qualitative screening tool for on-site rapid detection or epidemiological investigation in resource-limited settings. Under certain conditions, it may also be applied for semi-quantitative analysis; for example, by assessing the colorimetric intensity of the test strips across different concentration gradients, the bacterial load in samples can be roughly stratified (e.g., strong positive, moderate positive, weak positive, and negative), thereby meeting the requirements of preliminary clinical screening.

Regarding clinical sample validation, a total of 130 wild rodent spleen samples collected from five provincial-level administrative regions were tested in parallel using the RAA-LFD method, conventional PCR, and qPCR. The results of all three methods were completely concordant, with no positive signals detected in any sample. It should first be emphasized that positive controls were effectively amplified in each run, confirming the reliability and stability of the reaction system and the operational procedures. Under this premise, the uniformly negative results may be attributable to the following factors. First, although the sampling regions (Heilongjiang, Jilin, Liaoning, Sichuan, and Guangxi) cover parts of the natural plague foci in China, the sampling time may not have coincided with the peak epidemic season for plague (typically from September to April of the following year), and the sampling sites may not have precisely covered the core distribution areas of the primary host animals, such as the *Himalayan marmot* and the *Mongolian gerbil* [38]. The presence of *Y. pestis* in the natural environment exhibits pronounced ecological niche dependence and spatiotemporal heterogeneity, and its long-term survival is highly dependent on a closed-loop ecosystem comprising specific rodent hosts, flea vectors, and favorable geographical and climatic conditions [39]. Second, the pathogen load in some samples may have been below the method’s detection limit, or DNA degradation may have occurred during sample collection and transport. Although no positive samples were obtained in this clinical validation, the 100% concordance among the three methods still demonstrates the reliability of the RAA-LFD method in complex biological matrices, and its high consistency with routine laboratory molecular diagnostic methods provides important evidence for its application in primary surveillance networks.

Nevertheless, the method established in this study has certain limitations that warrant further improvement. First, as an open-tube endpoint detection method, RAA-LFD poses a risk of aerosol contamination during lateral flow strip reading. Second, the clinical validation samples in this study were all negative; thus, the method’s detection performance on authentic infected samples has not been adequately evaluated. Future studies should collect more samples from natural plague foci during the epidemic season or conduct validation using experimentally infected animal models or spiked samples. Lastly, the long-term stability of the detection reagents at ambient temperature and the batch-to-batch variability have not been systematically assessed, both of which are crucial for field deployment and commercial translation of the method.

## 5. Conclusions

In conclusion, this study successfully established a rapid detection method for RAA-LFD targeting the *pla* gene of *Y. pestis*. The assay can be completed within 30 min under isothermal conditions at 39 °C, with a limit of detection of 3.8 × 10^2^ copies/μL, excellent specificity, simple operation, and naked-eye visual interpretation of results.

## Figures and Tables

**Table 1:** Sequences of primers and a probe used in this study.

Target Gene	Direction	Primer and Probe ID	Sequence (5′–3′)	Amplicon Region (bp)
*p* *la*	Forward	P-F1	CTGAGTGGACAGATCACTCATCTCATCCTG	142
	Reverse	P-R1	5′Biotin-CTGAAACGTGTTTCCTGATATCCTGCTGT	
	Forward	P-F2	GATCACTCATCTCATCCTGCTACAAATGT	151
	Reverse	P-R2	5′Biotin-AACCACCTGTAGCTGTCCAACTGAAACGTG	
	Forward	P-F3	CTACAAATGTTAATCATGCCAATGAATATG	141
	Reverse	P-R3	5′Biotin-AACTATATGAACCACCTGTAGCTGTCCAAC	
	Probe	P-P	5′FAM-CCTCAATGTGAAAGGCTGGTTACTCCAGG [THF]TGGAATTATAAAGCAGGT-3′Spacer	

## Data Availability

The original contributions presented in this study are included in the article. Further inquiries can be directed to the corresponding authors.
